# Assessing hazard prediction and risk calibration skills in experienced and novice e-scooter riders

**DOI:** 10.1038/s41598-025-87538-y

**Published:** 2025-02-01

**Authors:** Petya Ventsislavova, Lydia Harrison, Thom Baguley

**Affiliations:** Nottingham Trent University, 50 Shakespeare St, NG1 4FQ Nottingham, USA

**Keywords:** E-scooters, Hazard prediction, Risk calibration, Riding experience, Decision-making, Psychology, Human behaviour

## Abstract

Less experienced e-scooter riders often exhibit risky riding behaviours. Despite this, no studies have examined how riders calibrate risk, respond to hazardous situations, and the impact of riding experience on these skills. To address this, this study assessed hazard prediction and risk calibration in e-scooter riders via bespoke video-based tests featuring real e-scooter footage filmed from the rider’s perspective. The first experiment assessed the ability of e-scooter riders to predict hazardous riding scenarios. The second experiment evaluated their proneness to engage in risky riding situations. The results indicated that increased riding experience did not improve riders’ hazard prediction skills or reduced their proneness to engage in risky riding. In fact, a higher riding frequency was linked to an increased tendency to engage in risky behaviour in certain scenarios. The results highlight that the typically short duration of e-scooter trips may limit riders’ exposure to a variety of hazards, hindering their ability to develop effective risk calibration skills. The observed high propensity to engage in risky riding scenarios, combined with average hazard prediction scores, emphasizes the need for targeted rider training focused on vigilance and risk awareness.

## Introduction

Electric scooters (e-scooters) were introduced to encourage a shift from individual car use to a more sustainable mode of transport. However, since their introduction into the transport system, numerous e-scooter collisions have been reported around the world^1^, which has led to a negative shift in public perception of these vehicles^2^. This has resulted in e-scooters being banned in various cities across Europe, and in the UK private e-scooters remain illegal^3^. However, these drastic and reactive measures have not provided a better understanding of how to reduce e-scooter collisions and improve micromobility safety. E-scooters have become emblematic of new technologies and design innovations within the micromobility sector, leading research to predominantly focus on enhancing the design and technical aspects of these vehicles^4,5^. However, this emphasis has often resulted in a relative neglect of the safety implications from a behavioural perspective. While studies have identified illegal and risky riding behaviour as one of the main causes of e-scooter collisions^6–8^, very few studies have explored e-scooter rider behaviour, and none have examined how riders calibrate risk and respond to hazardous situations.

Understanding riding behaviour is key to reducing collisions risk, as the UK Department for Transport (DfT) reported in 2022 that 40% of e-scooter collisions could be attributed to user error^9^, with 82% of injuries resulting from crashes affecting the e-scooter rider^10^. In addition, the DfT observed that a significant number of e-scooter collisions were reported by less experienced users. In fact, the DfT report indicated that first-time renters are about three times more likely to report a collision on their most recent trip than experienced users who have rented an e-scooter more than 20 times. This data suggests that a lack of adequate riding skills could also be a contributing factor to e-scooter collisions. Janikian et al.^7^ conducted a scoping review of 81 studies to identify the primary factors contributing to safety issues with e-scooters and equally identified rider experience as a significant behavioural crash contributor. Insufficient riding experience has also been linked to injury risk^11^.

Riding an e-scooter, and learning to do so safely, is a relatively new experience compared to more traditional modes of micromobility such as bikes. Research indicates that e-scooter riders are significantly more likely to engage in risky riding behaviour and are perceived by other road users as more reckless compared to cyclists or e-bike riders^12,13^. While multiple factors may contribute to e-scooter riders’ propensity for risky riding behaviour, their relatively limited riding experience compared to other modes of transport may result in insufficient skills for accurately calibrating risks and anticipating potential hazards while riding. At present, there is no evidence on riders’ ability to calibrate risk and identify hazardous scenarios, nor on the impact of riding experience on these skills.

In the cognitive transport literature, it is well-established that skills such as hazard awareness and the ability to read the road are crucial in preventing road collisions^14^. Hazard perception refers to a driver’s ability to identify potentially dangerous situations on the road with enough time to react and avoid a collision^15^. Research on hazard perception consistently demonstrates that safer, more experienced drivers show better ability to perceive hazards compared to inexperienced drivers^16–21^. Moreover, to effectively perceive hazards in time, safe drivers must possess the ability to anticipate them, allowing for adequate time to make informed and safe decisions rather than merely reacting to them^22,23^. Anticipating hazards takes precedence to hazard perception, as failure to direct attention to the right place at the right time can result in missing crucial cues necessary to avoid collisions. This process involves directing attention to relevant locations at the appropriate times, guided by top-down processes that anticipate hazards even before they occur^24^. The Biased Competition Theory posits that attention is selectively allocated to stimuli in the environment based on their relevance and significance^25^. Through repeated exposure and practice, experienced drivers develop heightened abilities to anticipate hazards using contextual cues and their past experience^26^. These drivers’ attentional biases enable them to efficiently detect and prioritise relevant stimuli amidst the complexities of the driving environment, leading to quicker and more accurate responses to potential hazards. In contrast, novice drivers often find predicting hazards more challenging due to less developed attentional biases and a lack of experience in recognising and responding to critical cues^27^.

It should be noted, however, that operating any vehicle safely is a complex cognitive task, where multiple tasks must be performed simultaneously^28^. As a result, hazard prediction alone may not guarantee risk and collision avoidance. To make appropriate decisions in dangerous scenarios, road users must also be able to adequately assess the associated risk that these scenarios involve in order to avoid the likelihood of a collision^29^. Risk calibration is a driver’s ability to accurately judge both the risk a dangerous situation poses and their ability to respond to it^30^. Research featuring video-based driving tasks has demonstrated that accuracy in driver estimates of the risk involved in potentially dangerous behaviours (e.g. speeding or driving through an amber light) predicts their likelihood to engage in these behaviours^31,32^. This has equally been observed in studies involving self-reported measures among e-bike riders where more accurate risk calibration while riding was predictive of a lower chance of engaging in risky riding behaviour^33,34^. The Risk Homeostasis Theory^30^ posits that the choice to engage in a specific risky behaviour is determined by comparing the perceived risk of that behaviour with the driver’s preferred level of risk, which may vary over time and according to different situations. When the perceived risk exceeds their preferred level, drivers adjust their behaviour to align the current level of risk with their personal tolerance. Consequently, it is anticipated that drivers will exercise greater caution on hazardous roads if they perceive them as dangerous and if the risk level exceeds their desired arousal level. As e-scooter riders seem to exhibit higher levels of risky riding behaviour compared to other road users^12^, evaluating their risk calibration abilities will provide insights into how they assess and adjust their risk levels in response to different risky riding conditions.

Similarly to hazard prediction, risk calibration skills have also been observed to improve with experience in the driving context, as novices typically show poorer risk calibration skills than their more experienced counterparts and are more likely to incorrectly rate dangerous situations as low risk^18,32,35–37^. However, defining what constitutes an experienced e-scooter rider is challenging due to the novelty of these vehicles and diverse usage patterns of e-scooter riders. E-scooters were introduced in Europe relatively recently, between 2018 and 2020, limiting the opportunity for even the most dedicated riders to develop the level of experience typically acquired with more established forms of transportation, such as cars or bicycles. Furthermore, e-scooters are typically used for short journeys, with riders often being either one-time or irregular users^38^. In contrast, the definition of an experienced driver or cyclist is more clearly established, with studies indicating that years of practice and accumulated mileage are reliable indicators of driving or riding experience^39–41^.

Some studies have attempted to quantify e-scooter experience by measuring the frequency of e-scooter usage or ownership. For instance, Uluk et al.^42^ conducted a study investigating risk factors in e-scooter injuries, including the role of experience. They recruited participants from a hospital emergency department who were admitted due to e-scooter-related injuries and defined an experienced rider as any individual who had previously ridden an e-scooter prior to the incident that led to their admission. Other studies have measured experience by asking participants about the frequency with which they use e-scooters^43^ or the number of journeys made (e.g., five or more rides to qualify as an experienced rider)^44^. However, no studies have accounted for the duration of time since a rider began using an e-scooter, such as months/years of riding experience. A rider may be frequent, but if they have only been using their e-scooter for a month, they could arguably have the same amount of experience as someone who rides less frequently but over a period of 12 months. As a result, in this study, we considered both factors—years/months of riding experience and frequency of riding—to develop a more robust definition of riding experience. This approach offers a more precise measure than simply asking participants if they have ridden an e-scooter in the past.

### The present study

Considering that the ability to predict hazards and calibrate risk is negatively correlated with collision risk in driving and cycling, it was hypothesised that these skills would also be crucial for safe e-scooter riding. Since higher experience with other vehicles appears to influence both skills, this study aimed to assess whether experienced e-scooter riders would demonstrate superior hazard prediction and risk calibration skills in comparison to novice riders. Experience in e-scooter riding was defined by extrapolating parameters from studies on hazard prediction and risk calibration in car drivers, considering factors such as mileage, usage frequency, and length of experience^22,29,45^. To the best of the authors’ knowledge, no previous studies have specifically evaluated these skills in e-scooter riders, nor has the link between these skills and riding experience been explored to assess whether riding safety improves with experience.

To assess these skills, bespoke video-based hazard prediction and risk calibration tests were developed featuring real riding footage of hazardous and risky scenarios. Previous variants of the hazard prediction test have already proven to be highly effective in distinguishing between experienced and novice drivers^22,27^, cyclists^46^, e-bike riders^47^, and motorcyclists^48^. Similarly, the risk calibration test, which follows a comparable video-based format, has also been demonstrated to effectively differentiate between experienced and novice riders^29,32^. For this study, both tests were adapted to feature hazardous and risky scenarios from the perspective of an e-scooter rider.

It was hypothesised that experienced riders would show a better ability to predict hazardous situations and would be less prone to engage in risky riding behaviour compared to novices. It was also expected that both the frequency of riding and the number of months of experience would be equally predictive of good hazard and risk calibration skills, with neither factor demonstrating a clear advantage over the other. Finally, it was expected that experienced riders would exhibit a reduced propensity for risk-taking compared to novices, with this tendency varying by the type of risky scenario.

## Methods

### Design

The study employed a mixed design, incorporating both between-subjects and within-subjects components. The between-subjects component focused on the level of riding experience, and the within-subjects component involved the risky scenario for the risk calibration test. All participants were assessed separately across two independent domains: hazard prediction and risk calibration. The risk calibration assessment included three different types of riding scenario, where participants were asked to make decisions on their riding behaviour for situations involving traffic lights, overtaking, and speeding. The hazard prediction task did not include hazard categories but instead evaluated participants’ overall ability to predict a variety of hazards.

Riding experience was defined according to months of riding experience and frequency of riding. While participants’ yearly riding mileage was also assessed, this was not included in the final model due to the inability of many of the participants to provide an accurate estimate of miles ridden. As e-scooters are primarily used for short trips and do not display miles ridden or require refuelling, estimating accurate mileage can be more challenging compared to cars. Nevertheless, we have included estimated mileage data in Table 1. While participants reported between six months and three years of riding experience, such length is typically representative of novice drivers in the driving context. In other words, it could be argued that all riders are essentially novices. However, given the unique nature of e-scooters and their novelty, three years of riding experience could represent an experienced rider in comparison to someone who has used an e-scooter for six months. In order to account for this, the duration of riding experience (measured in months) was paired with frequency of use to ensure that those riders who report more than one year of riding experience also ride frequently.

The independent variables were months of riding experience and frequency of riding, as well as the type of risky riding scenarios for risk calibration. The dependent variables were accuracy in hazard prediction and likelihood to engage in risky riding.

### Participants

In total, 244 participants were recruited for this study, however 57 participants were subsequently excluded. This was due to either missing data (*n* = 29); inconsistent or incomplete responses as to their e-scooter usage (*n* = 7); never used an e-scooter (*n* = 16); selecting “N/A” when giving their frequency of e-scooter usage (*n* = 5). Of the final 187 participants included in the study, 102 participants indicated they were female, 81 indicated they were male, 2 indicated they were non-binary and 2 indicated they preferred not to say. Participants had an average age of 34.50 (*SD* = 11.76), ranging from 19 to 73, excluding one participant who declined to report their age. The overall demographic information and riding experience for these participants is outlined in Table 1.

Participants were recruited online through the recruitment platform Prolific (www.prolific.com) and were compensated in line with Prolific’s policy, which is approximately £9 per hour for their participation in the experiment.

**Table 1:** Demographic and riding information of the participants across frequency of e-scooter usage.

	Daily	Weekly	Monthly	Once every 6 months	Once every 12 months
N Participants	15	47	39	50	36
Average age	32.13	32.76	36.49	35.56	34.08
Age *SD*	10.04	11.60	14.53	11.68	9.19
N Gender					
Male	10	24	15	20	12
Female	5	22	23	29	23
Non-Binary				1	1
Prefer not to say		1	1		
Estimated miles travelled per year	Daily	Weekly	Monthly	Once every 6 months	Once every 12 months
Mean	1866.87	1371.13	190.56	17.61	7.40
SD	2344.40	2110.73	794.74	26.34	8.59
Estimated length of riding experience in months	Daily	Weekly	Monthly	Once every 6 months	Once every 12 months
Mean	21.40	22.62	18.72	17.18	14.97
SD	14.14	13.82	15.63	10.49	13.89

### Materials

To develop the hazard prediction and risk calibration tests, bespoke video stimuli were created and edited into hazardous and risky video clips. The process of generating the video stimuli has been described below.

#### Filming of the video footage

**Fig. 1:**
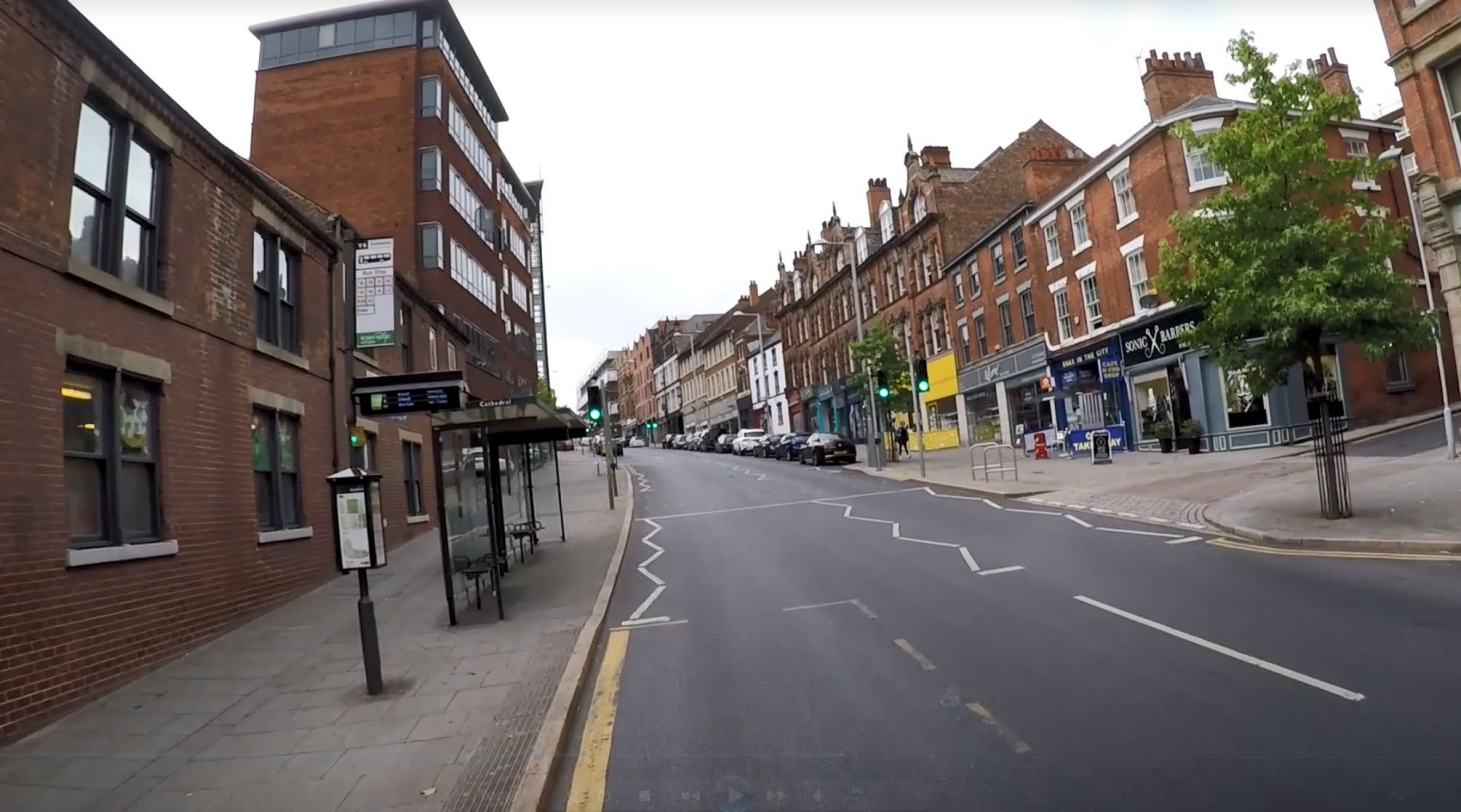
A screenshot of a riding scenario filmed from the perspective of an e-scooter rider and part of the video footage. Please note that the image is slightly tilted because the camera is mounted on the rider’s helmet, which moves with their head and therefore does not always capture a straight angle.

The video footage was recorded during normal e-scooter riding around Nottingham and the surrounding suburban areas. The footage was filmed following the Health and Safety guidelines for filming from moving vehicles by Nottingham Trent University. A rider with four years of riding experience captured the footage. E-scooter rules and national regulations were followed during the entire filming process and only e-scooters which were part of the public trial were used for the filming process. None of the hazardous scenarios captured during the filming were staged. The footage was captured from the rider’s perspective using a GoPro HERO3 camera mounted on the e-scooter rider’s helmet (see Fig. 1). The camera did not obstruct the view of the e-scooter rider or present any danger to pedestrians or other road users. The footage was recorded using a resolution of 1920 × 1080 and a frame rate of 59.94fps.

Following recording, the video footage was edited into short video clips via the video editing software Adobe Premier Pro. The entire footage was reviewed frame by frame to identify potential hazardous and risky scenarios that could be included as part of the risk and hazard tests. Two types of video categories were generated: videos where riders were asked to predict hazardous situations and videos where riders were asked to calibrate the risk each scenario involves and make a decision on how to proceed. For instance, the clips generated for the hazard prediction test included scenarios that were occluded just prior to a hazardous situation and participants were asked to predict what would happen next in the riding scene (e.g. a pedestrian about to cross the road). The clips generated for the risk calibration test reflected situations where participants were required to decide the likelihood of them engaging in a specific risky scenario (e.g. ride through a traffic light in red).

All clips for all tests were edited to occlude prior to the hazardous/risky scenario unfolded to prevent participants from observing how the rider filming the footage actually proceeded in each situation. Each occlusion was followed by a black screen to prevent the clip from freezing on a final frame that might reveal the rider’s action or hazard. A freeze-frame provides an unrealistic amount of time for identifying clues to impending hazards compared to real life, whereas an occlusion ensures that riders must focus on the correct area at the right moment. Since safer riders are more likely to prioritise areas of the scene that may develop into hazards, the occlusion is, therefore, more likely to identify the safest riders^27,49^. The occlusion points for each clip were determined following an empirical protocol for developing hazard prediction clips (see Ventsislavova & Crundall^50^ for more information on the method used to select the occlusion points). It is crucial to select the optimal points for occluding the clips, ensuring each clip provides sufficient clues for effective accuracy in responding to the selected situations. The selection of scenarios and the final occlusion points were evaluated and approved by expert traffic psychologists and subsequently piloted. The process of selecting the video clips for each test has been described in the next section.

#### Video clips selection

A total of 23 video clips were included across the two tests. The hazard prediction test included 12 clips and the risk calibration test included 11clips.

##### Hazard prediction clips

**Fig. 2:**
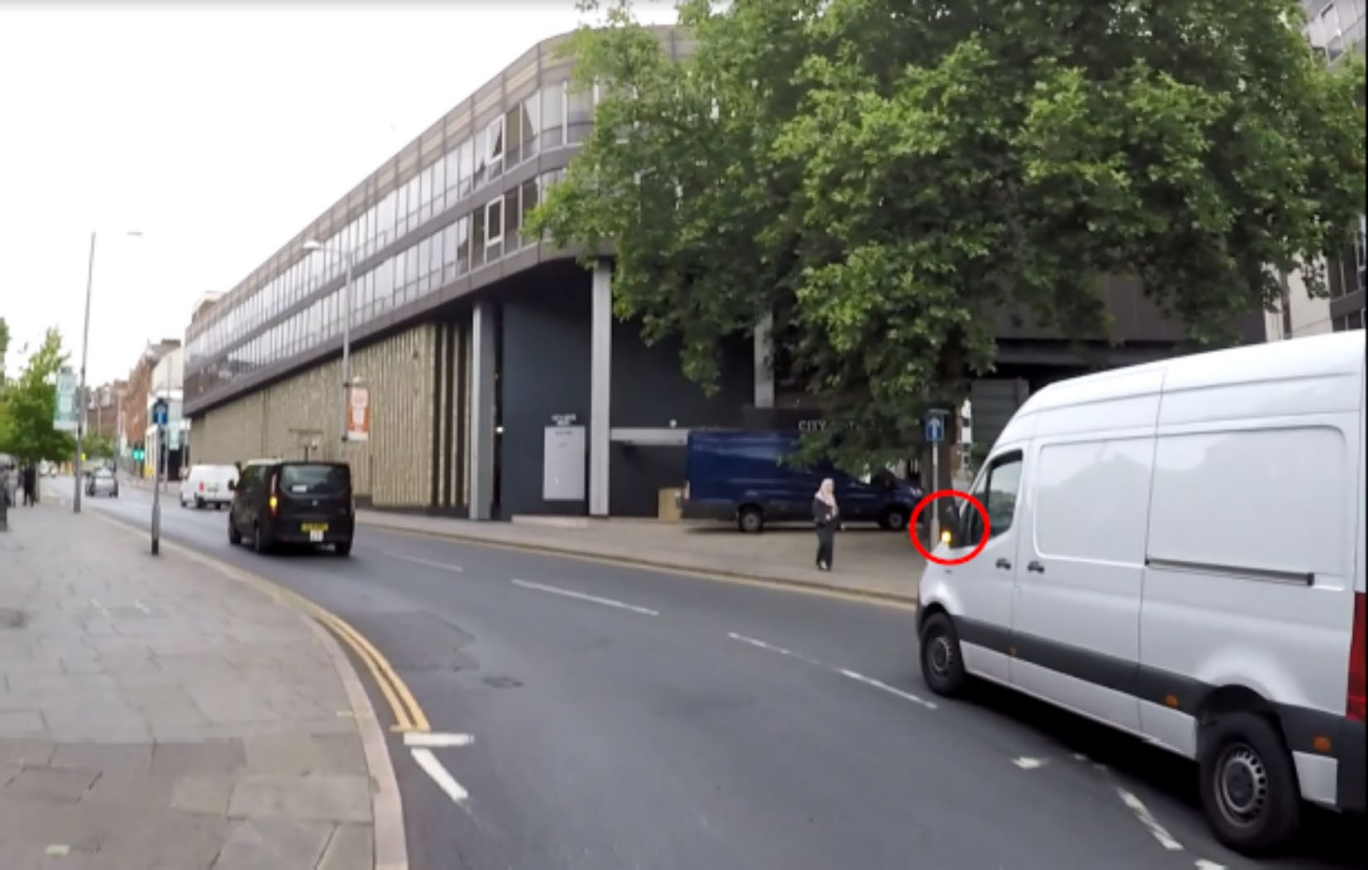
The image captures the moment just prior to occlusion, where a white van is overtaking the e-scooter rider closely and signalling to merge into the rider’s lane. If the rider misses this cue, (car’s indicator circled in red), they may be unable to anticipate the van driver’s intention.

The hazard prediction clips included a variety of hazardous scenarios – for example pedestrians, overtaking cars, cyclists or cars cutting the rider’s path (see Table 2 for a detailed description of each hazard). Each one of the video clips included materialised hazards rather than potential ones (hazard that did happen vs. hazards that could have happened but did not). For example, one of the hazardous scenarios involved a van closely overtaking the e-scooter rider and then signalling to merge into the rider’s lane. If the rider were to miss the car’s indicator (precursor to the hazard), they might not be able to anticipate the van driver’s intention (see Fig. 2). If the clip had continued, participants would have observed the van cutting into the rider’s path. Following occlusion, participants were asked to predict the hazardous situation by choosing one of four possible options. This multiple-choice format is commonly used in hazard prediction tests and has been demonstrated to be more sensitive to driving experience compared to an open response format^50^. For this format, the plausibility of the distractors is crucial to maintaining the effectiveness of the test, as it prevents the correct answer from being too obvious. For this end, guidance from Worthen et al.^51^ and Haladyna et al.^52^ was followed, wherein incorrect options were generated based on responses obtained from 10 participants during a pilot study. This pilot study was conducted specifically to obtain potential distractors for the hazard prediction video clips. Participants were asked to provide written responses to the 12 clips, with their incorrect answers used as distractors. This approach has been successfully employed in previous hazard prediction studies^23,53,54^. In addition, the generated options were reviewed by a team of transport psychology experts and subsequently piloted (see “Pilot conducted to validate the video clips” section). Finally, the occlusion points were selected following the methodology of Ventsislavova & Crundall^50^ for developing hazard prediction tests.

**Table 2:** A description of the hazardous video clips presented to participants during the hazard prediction task, as well as the precursors participants should have been attending to, who the scenario posed the greatest risk to and the correct safe action to take.

Clip 1	
Description	The e-scooter rider is driving up a hill in the bus lane. Part of the road is obstructed by road works. The e-scooter rider approaches a side street, turning their head to check if they can move lanes. A white car begins to overtake the e-scooter on the rider’s right
Precursors present within the clip	The white car becomes visible just prior to the clip occlusion
Who is at risk	If the e-scooter rider did not perceive this hazard, they are at risk of a side-on collision. The greatest risk is posed to the e-scooter rider
Correct action to be taken	The e-scooter rider should slow down and not change lanes
Clip 2	
Description	The e-scooter rider is turning onto a cycle lane and riding up a hill. The e-scooter rider passes through traffic lights and continues up the hill. A van on the right-hand side of the lane becomes visible, and a light additionally becomes visible directly ahead of the e-scooter rider on the cycle lane. This indicates the presence of another road user ahead, who is riding down the centre of the cycle path.
Precursors present within the clip	The light ahead of the e-scooter rider – in this instance, another e-scooter rider – is present in the final second of the video clip.
Who is at risk	An inexperienced e-scooter rider may find themselves distracted by the van on the other side of the road, which poses no threat to them. If both e-scooter riders were to miss this hazard, they are at risk of a head-on collision. Alternatively, a last-minute evasive reaction could cause the e-scooter rider to lose balance. Both e-scooter riders are at risk of injury
Correct action to be taken	The e-scooter rider should slow down and manoeuvre themselves closer to left to avoid the oncoming e-scooter.
Clip 3	
Description	The e-scooter rider is at a red light on a four-way intersection. As the light turns green, the e-scooter rider pulls out and turns right, riding up a hill in a bus lane. The e-scooter rider is passed on their left by a van, followed by a car which begins to indicate that they are turning left, and will cut the e-scooter rider’s path.
Precursors present within the clip	The participant is shown the cars’ indicator blinking once. This is in line with the amount of information presented in similar hazard stimuli created for the study of hazard prediction in driving.
Who is at risk	The car is indicating that they are going to pull into the e-scooter rider’s path. If the e-scooter doesn’t break in time, they are at risk of colliding with the rear or side of the car. Alternatively, a last-minute evasive reaction could cause the e-scooter rider to lose balance. Only the e-scooter rider is at risk of injury
Correct action to be taken	The e-scooter rider needs to slow down in a timely manner and allow the grey car to complete their manoeuvre.
Clip 4	
Description	The e-scooter rider bears left and rides down a high street in the bus lane. They pass a set of lights and a side road. The bus lane ends, and the road is lined with parked cars on either side. The e-scooter rider pulls to the right and continues riding in the road. A pedestrian steps off of the pavement and between two parked cars. They look both ways, indicating their intention to cross the street.
Precursors present within the clip	An experienced e-scooter rider should be aware that parked cars can conceal hidden pedestrians waiting to cross. In this instance, the pedestrian’s movement towards the pavement are visible for the final two seconds of the clip and their body language clearly indicates their intention to cross.
Who is at risk	If the e-scooter rider did not see the pedestrian in time, or if the pedestrian steps out having not seen the e-scooter rider, they are at risk of colliding with each other. This poses a risk of injury both to the pedestrian and the e-scooter rider
Correct action to be taken	The e-scooter rider should slow down as they pass the pedestrian; to lower the risk of injury should the pedestrian step out in front of them
Clip 5	
Description	The e-scooter rider is driving through a high street. They attempt to ride in the bus lane, however the presence of vehicles incorrectly parked here require the e-scooter rider to pull further across to the right. They continue to ride through the lights and are passed on their right by a car. While the e-scooters side of the road is quiet, the oncoming traffic on the right-hand side is busy. The e-scooter rider passes through a second set of lights and is approaching a side street on their left. On the right-hand side of the road, an oncoming van begins to indicate their intention to turn into this side road and begins to position themselves to turn
Precursors present within the clip	The positioning of the van clearly indicates their intention to turn into the side road, and therefore cut the e-scooter rider’s path. Additionally, the van signals with their right indicator twice before the clips end
Who is at risk	If the e-scooter rider fails to observe the van turning into their path in a timely manner, they are at risk of colliding with this vehicle. This poses significant risk to the e-scooter rider, depending upon both their own speed and the speed of the van
Correct action to be taken	The e-scooter rider should slow their speed, and potentially stop, to allow the van to complete their manoeuvre.
Clip 6	
Description	The e-scooter rider is travelling down a busy road and bears left. They continue to ride forwards towards a convenience store positioned on the corner of a narrow side street. The e-scooter rider bears left again down this side street. Further down this road a black car driving in the opposite direction to the e-scooter rider becomes visible
Precursors present within the clip	An experienced rider should be aware of the potential for oncoming traffic down a street of this nature. The black car is visible for the final 40 frames of the clip. Its lights are on, and it is moving.
Who is at risk	If the e-scooter rider was to come round this corner at a high speed or if the e-scooter rider was to notice this vehicle too late, the rider is at risk of a head on collision. This poses a risk of injury to the e-scooter rider.
Correct action to be taken	The e-scooter rider should slow, or potentially stop, to allow the vehicle to pass them safely.
Clip 7	
Description	The e-scooter rider passes a park on their right, then rides straight across a four-way intersection. The e-scooter rider is approaching a tram stop and a set of lights ahead. A pedestrian exits a store on the e-scooter riders’ left and heads towards the road, indicating their intention to cross the road and therefore cut the e-scooter riders’ path.
Precursors present within the clip	The pedestrian is visible in the final second of the stimuli, and from their body language it is clear that the pedestrian intends to cross
Who is at risk	If the e-scooter rider was going too fast, or if the pedestrian was to cross without looking, they are at risk of a collision. This would pose a risk of injury both to the pedestrian and to the e-scooter rider.
Correct action to be taken	The e-scooter rider should slow their speed, and bear to the right to avoid the pedestrian.
Clip 8	
Description	The e-scooter rider is stopped by a red light. As the lights change, they ride through a four-way intersection and bear left. The e-scooter rider mounts the pavement to avoid a tram parked at a tram stop then slows to allow a pedestrian to pass, then begin to pick up speed again. An oncoming pedestrian in a mobility scooter appears from behind a lamp post.
Precursors present within the clip	The mobility scooter is present for the final 34 frames of the stimuli. As the e-scooter rider has mounted the pavement, they should be aware of the potential that there will be oncoming pedestrians obscured. Additionally, the mobility scooter is in motion and is a bright orange, making it more visually salient
Who is at risk	If the e-scooter rider failed to observe the oncoming mobility scooter, they are at risk of collision. Alternatively, a last-minute evasive reaction could cause the e-scooter rider to lose balance. Both the e-scooter rider and the pedestrians present within the stimuli are at risk of injury
Correct action to be taken	The e-scooter rider should stop and dismount their e-scooter.
Clip 9	
Description	The e-scooter rider is riding through a residential area. Cars are parked along both the left and right sides of the road. The e-scooter rider passes a side street and continues to ride straight, positioning themselves in a cycle lane. At the end of the street, scaffolding becomes visible behind the rows of parked cars. The e-scooter rider passes a parked van, and a pedestrian in a high-vis jacket becomes visible. The pedestrian steps towards the road, intending to cut the e-scooter riders’ path
Precursors present within the clip	The pedestrian is visible for the last 1 s and 6 frames of the stimuli, and their body language makes their intention to cross the road clear. Moreover, the presence of scaffolding within the stimuli should alert an experienced rider to the likelihood that pedestrians at work are nearby.
Who is at risk	If the e-scooter rider did not observe the pedestrian in time, they are at risk of collision. Alternatively, a last-minute evasive reaction could cause the e-scooter rider to lose balance. Both the e-scooter rider and the pedestrians present within the stimuli are at risk of injury
Correct action to be taken	The e-scooter rider should have slowed their approach on noticing the scaffolding ahead.
Clip 10	
Description	The e-scooter rider is at a red light at a busy roundabout. As the light goes green, they ride forward, keeping to the left. Several cars pass the e-scooter rider on the right, including a taxi which signals and pulls into the e-scooter riders’ lane. The e-scooter rider exits the roundabout and begins to bear left up a high street. A white van on the e-scooter riders right begins to signal their intention to turn left.
Precursors present within the clip	The white van signals with their indicator that they intend to cut the e-scooter riders’ path and join their lane. The indicator blinks once, which is in line with the amount of information presented in similar hazard stimuli created for the study of hazard prediction in driving.
Who is at risk	If the e-scooter rider fails to observe the white vans’ manoeuvre, they are at risk of colliding with this vehicle. This places the e-scooter rider at risk of injury.
Correct action to be taken	The e-scooter rider should slow, and potentially stop, to allow the van to finish its manoeuvre safely.
Clip 11	
Description	The e-scooter rider is exiting a busy roundabout and enters a busy commercial street. They are positioned in the right-most lane, however, begins to move left with the intention of joining the bus lane. As they move into the middle lane, a bus becomes visible ahead of them. This bus begins to signal and starts to join the middle lane, cutting the e-scooter riders’ path
Precursors present within the clip	The stimuli include the bus’s indicator blinking once, and the positioning of the bus clearly indicates the buses intention to pull into the middle lane.
Who is at risk	If the e-scooter rider failed to observe the buses intention, they are at risk of colliding with the back of the bus. Further, as this is a busy commercial area, if the e-scooter rider was to fall from their vehicle they are at risk of injury from the vehicles behind them.
Correct action to be taken	The e-scooter rider should slow, and potentially stop, to allow the bus to finish its manoeuvre safely.
Clip 12	
Description	The e-scooter rider is riding through a residential area on the cycle lane. Ahead of the e-scooter rider a pedestrian crosses the road. The e-scooter rider passes this pedestrian and begins to approach a side street on the e-scooter riders left. The e-scooter rider passes the side street, and ahead a pedestrian becomes visible angled towards the cycle lane, indicating their intention to cross
Precursors present within the clip	As the e-scooter rider is in a residential area, an experienced rider would know to expect pedestrians. The pedestrian in the clip is present and indicating their intention to cross.
Who is at risk	If the e-scooter rider did not observe the pedestrian in time, they are at risk of collision. Alternatively, a last-minute evasive reaction could cause the e-scooter rider to lose balance. Both the e-scooter rider and the pedestrians present within the stimuli are at risk of injury
Correct action to be taken	The e-scooter rider should slow their speed, to ensure that if the pedestrian was to step out in front of them, they would have the time to respond safely.

##### Risk calibration clips

**Fig. 3:**
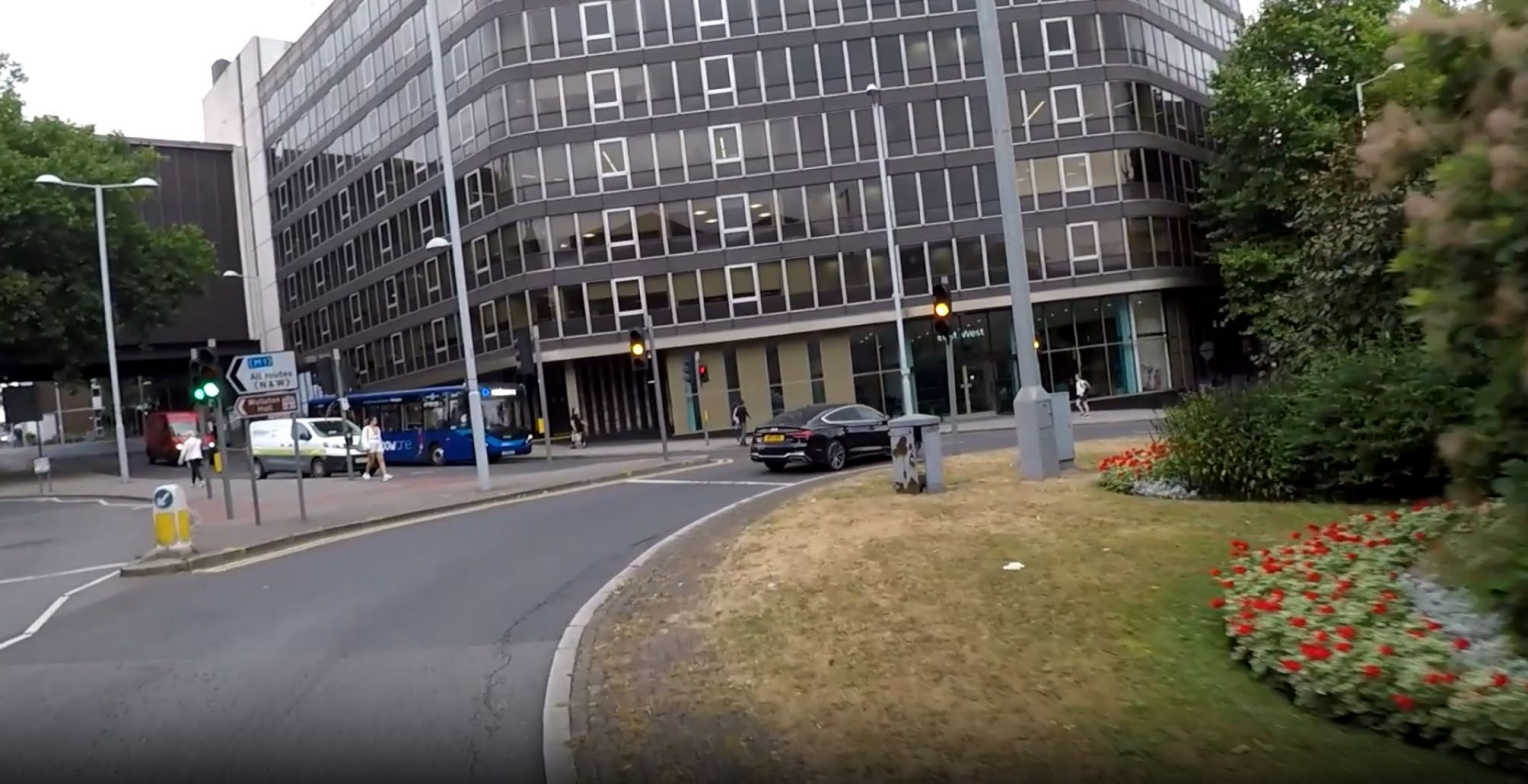
The image depicts the perspective of an e-scooter rider approaching a traffic light that has just turned amber. This is the point just prior to occlusion, where participants are asked to assess their likelihood of proceeding through the amber light.

In the risk calibration task, the selected scenarios featured dilemmas involving traffic lights, as well as situations that required overtaking and speeding decisions. Each clip depicted a situation where the rider was required to decide whether or not to proceed during a risky situation. Three of the clips included scenarios where participants had to determine if it was safe to overtake a vehicle on the road. For instance, one scenario involved a vehicle on a 30-mph road with cars driving both behind the e-scooter rider and approaching from the opposite direction on a single carriageway. The video was occluded just prior to the rider’s action and participants were asked to rate from 1 to 7 (1- very unlikely and 7 – very likely), “How likely would you be in this situation to overtake the parked car and continue riding”. Three of the clips included speeding decisions. Participants were asked to rate from 1 to 7 how likely they would be to ride at 20mph in each of the scenarios considering the legal speed limit for e-scooters. Finally, five clips included traffic light situations. Three of the clips included scenarios with red lights and two with amber lights. Each video was occluded just as the rider approached the traffic lights, requiring participants to rate their likelihood of continuing to ride through the lights in amber or red. For instance, in one scenario, the rider was slowly approaching a green light that changed to amber. The video was occluded at the moment the light turned amber, prompting participants to rate how likely they would be to continue through the light in amber (see Fig. 3).

The correct response in all scenarios, according to the rules for both drivers and riders, would always be to stop for the traffic lights and avoid overtaking or speeding. Participants were instructed to consider their knowledge of current UK e-scooter legislation when responding to the video clips. The decision for occlusion points followed the methodology of Ventsislavova et al.^32^, who developed a similar risk calibration task for car drivers.

It should be highlighted that none of the selected scenarios involving overtaking, amber lights, or speeding were safe for the rider to action. The scenarios were selected and judged by a panel of transport psychologists who are experts in the field. While there may be instances where overtaking a bus or cyclist can be safe (for example, when the road is clear ahead and there is sufficient distance from the other road user without exceeding the speed limit), performing these actions in the selected scenarios would be considered as dangerous (see Table 3 for a detailed description of each risky situation). Consequently, none of the selected scenarios presented a safe opportunity to proceed or assume the associated risks.

**Table 3:** A description of the risky video clips included within the risk calibration task, including the scenario type, a description of who is at risk in the clip and why and what the correct course of action would be.

Clip 1	
Scenario type	Speed
Description	The e-scooter rider is riding down a busy residential area. They pass a school on their left as well as a sign that indicates that the road has a speed limit of 20mph. The rider continues straight, passing a side street on their left where a car is pulling out.
Who is at risk?	If the e-scooter rider chooses to rider over the legal limit for an e-scooter rider (at the time of this recording, 15.5mph) then they are increasing their own risk of injury if they were to experience a collision, compared to riding at slower speeds. Additionally, if a pedestrian was to block their path, which is a significant risk given the school, they would increase the likelihood of a severe injury by colliding with them at a greater speed
Correct course of action	The e-scooter rider should ride within the legal speed limits for e-scooters, and therefore not exceed 15.5mph.
Clip 2	
Scenario type	Speed
Description	The e-scooter rider is travelling through a residential area. Parked cars line the street to the e-scooter rider’s left, and the e-scooter rider passes two side streets on their right and checks for the presence of oncoming cars before passing.
Who is at risk?	As this is a residential area, and the pavements are partially obscured by parked cars, an inexperienced e-scooter rider is at risk of collision with a pedestrian stepping into the road, and speeding would reduce their ability to react to this hazard in a timely manner. This places both the e-scooter rider and any potential pedestrians at risk of injury
Correct course of action	The e-scooter rider should ride within the legal speed limits for e-scooters, and therefore not exceed 15.5mph.
Clip 3	
Scenario type	Speed
Description	The e-scooter rider is travelling down a quiet residential area. As they travel, road markings become visible that indicate the speed limit for this street is 20mph. The e-scooter rider passes a side street on their left, and is approaching a junction at the end of the clip
Who is at risk?	If the e-scooter rider chooses to rider over the legal limit for an e-scooter rider then they are increasing their own risk of injury if they were to experience a collision, compared to riding at slower speeds. Additionally, as this is a residential area, they are at risk of colliding with a pedestrian if they travel at too greater speeds to react in a timely manner.
Correct course of action	The e-scooter rider should ride within the legal speed limits for e-scooters, and therefore not exceed 15.5mph.
Clip 4	
Scenario type	Overtaking
Description	The e-scooter rider is travelling down a busy high street, with parked cars lining both streets. They intend to ride in the bus lane; however, a car has incorrectly parked here, and the e-scooter rider must choose what to do as a result.
Who is at risk?	Depending on the actions of the rider, there are several risks present. A rider who chooses to pull further to the right and overtake the car in this manner must check behind them before doing so. If they did not, they are at risk of collision from potential vehicles behind them. If the e-scooter rider chooses to pass the parked car on the left, and they do not dismount their e-scooter, they are at risk of collision with pedestrians who are obscured by the parked car on the pavement, placing both the e-scooter rider and the pedestrian at risk.
Correct course of action	The e-scooter rider firstly should slow down and stop to look. If they decide to remain in the road, they should check the traffic behind them to ensure they do not ride into the path of an oncoming vehicle. If they choose to exit the road, they must stop their e-scooter, dismount and walk with their e-scooter until they re-enter the road.
Clip 5	
Scenario type	Overtaking
Description	The e-scooter rider is riding through a busy residential area, approaching a set of lights. They are overtaken by a van and pass a side street. As they do so, they approach a car parked on the left, as well as a van with flashing lights further down the road, indicating the presence of roadworks.
Who is at risk?	Depending on the actions of the rider, there are several risks present. A rider who chooses to pull further to the right and overtake the car in this manner must check behind them before doing so. If they did not slow down and check, they are at risk of collision from potential vehicles behind them. Similarly, given the presence of the road works, they must be vigilant of the risk of workers in the road following their overtaking manoeuvre. If the e-scooter rider chooses to pass the parked car on the left, and they do not dismount their e-scooter, they are at risk of collision with pedestrians or workers who are obscured by the parked cars and the van, placing both the e-scooter rider and the pedestrians at risk.
Correct course of action	The e-scooter rider firstly should slow down. If they decide to remain in the road, they should check the traffic behind them to ensure they do not ride into the path of an oncoming vehicle and maintain their slower speed to ensure they do not collide with workers in the road. If they choose to exit the road, they must stop their e-scooter, dismount and walk with their e-scooter until they re-enter the road.
Clip 6	
Scenario type	Overtaking
Description	The e-scooter rider is travelling down a residential street. Both sides of the street are lined with parked cars, and at the beginning of the clip an oncoming car signals their intention to park on the right. The e-scooter rider passes a side street and enters a cycle lane. They approach a van with open back doors parked within the cycle lane.
Who is at risk?	Depending on the actions of the rider, there are several risks present. A rider who chooses to pull further to the right and overtake the van must check behind them before doing so. If they did not, they are at risk of collision from potential vehicles behind them. Similarly, given the open doors of the van, this suggests the presence of pedestrians around the vehicle that the e-scooter rider needs to be aware of to prevent a collision.
Correct course of action	The e-scooter rider firstly should slow down. If they decide to remain in the road, they should check the traffic behind them to ensure they do not ride into the path of an oncoming vehicle and maintain their slower speed to ensure they do not collide with any pedestrians in the road. If they choose to exit the road, they must stop their e-scooter, dismount and walk with their e-scooter until they re-enter the road.
Clip 7	
Scenario type	Traffic lights
Description	The e-scooter rider exits a cycle lane and enters a road heading towards a roundabout. They approach a set of traffic lights where pedestrians are currently crossing the road, and the traffic lights are red
Who is at risk?	The e-scooter rider is at risk of severe injury if they choose to run the red light, as they may become involved in a collision with a car on the roundabout who was not expecting them. Additionally, as pedestrians are currently crossing the red light, if the e-scooter rider chose to run the red light they are putting the pedestrians at risk of a collision and therefore injury.
Correct course of action	The e-scooter rider should slow and stop in the bike box.
Clip 8	
Scenario type	Traffic lights
Description	The e-scooter rider is travelling down a bus lane, approaching a set of traffic lights. These lights are green, and the e-scooter rider therefore rides through these, turning left. As the e-scooter rider turns left, they approach a second set of lights which are red. Past the red lights, vehicles can be seen joining the road from the right
Who is at risk?	If the e-scooter rider was to run the red light, they would be putting themselves at risk of severe injury from vehicles joining this road who do not expect an e-scooter rider to be in the road at that moment.
Correct course of action	The e-scooter rider should slow and stop at the red light.
Clip 9	
Scenario type	Traffic lights
Description	The e-scooter rider is riding through a busy commercial area, approaching a set of traffic lights ahead of a roundabout. On approach, the lights are green, however as the e-scooter rider approaches these turn amber.
Who is at risk?	If the e-scooter rider continues to travel at their current speed, they may increase their risk of colliding with an oncoming vehicle currently on the roundabout
Correct course of action	The e-scooter rider should slow down and stop in the bike box.
Clip 10	
Scenario type	Traffic lights
Description	An e-scooter rider is travelling around a large roundabout, in the right-most lane. As they travel, they approach a set of traffic lights which are amber.
Who is at risk?	If the e-scooter rider continues to travel at their current speed, they may increase their risk of colliding with an oncoming vehicle currently on the roundabout who does not expect to be vigilant for e-scooter riders
Correct course of action	The e-scooter rider should slow down and stop at the lights.
Clip 11	
Scenario type	Traffic lights
Description	The e-scooter rider is waiting at a crossing. They begin to cross the road and position themselves to turn right onto a road which also contains tram tracks. Ahead of them, a red traffic light is clearly visible.
Who is at risk?	If the e-scooter rider was to run the red light, they would be putting themselves at risk of severe injury from vehicles joining this road who do not expect an e-scooter rider to be in the road at that moment. Additionally, as this road serves both cars and trams, by running the red light they are putting themselves at risk of colliding with a tram turning the corner.
Correct course of action	The e-scooter rider should stop and wait for the light to turn green before joining the road.

#### Pilot conducted to validate the video clips

The initial selection of video clips for all three tests was piloted with nine participants. The purpose of this pilot was to verify that participants could accurately understand the hazardous or risky scenarios presented, understand the instructions provided, and correctly interpret the response options. Based on the pilot feedback, adjustments were made to any clips or response options that were unclear to participants.

**Fig. 4:**
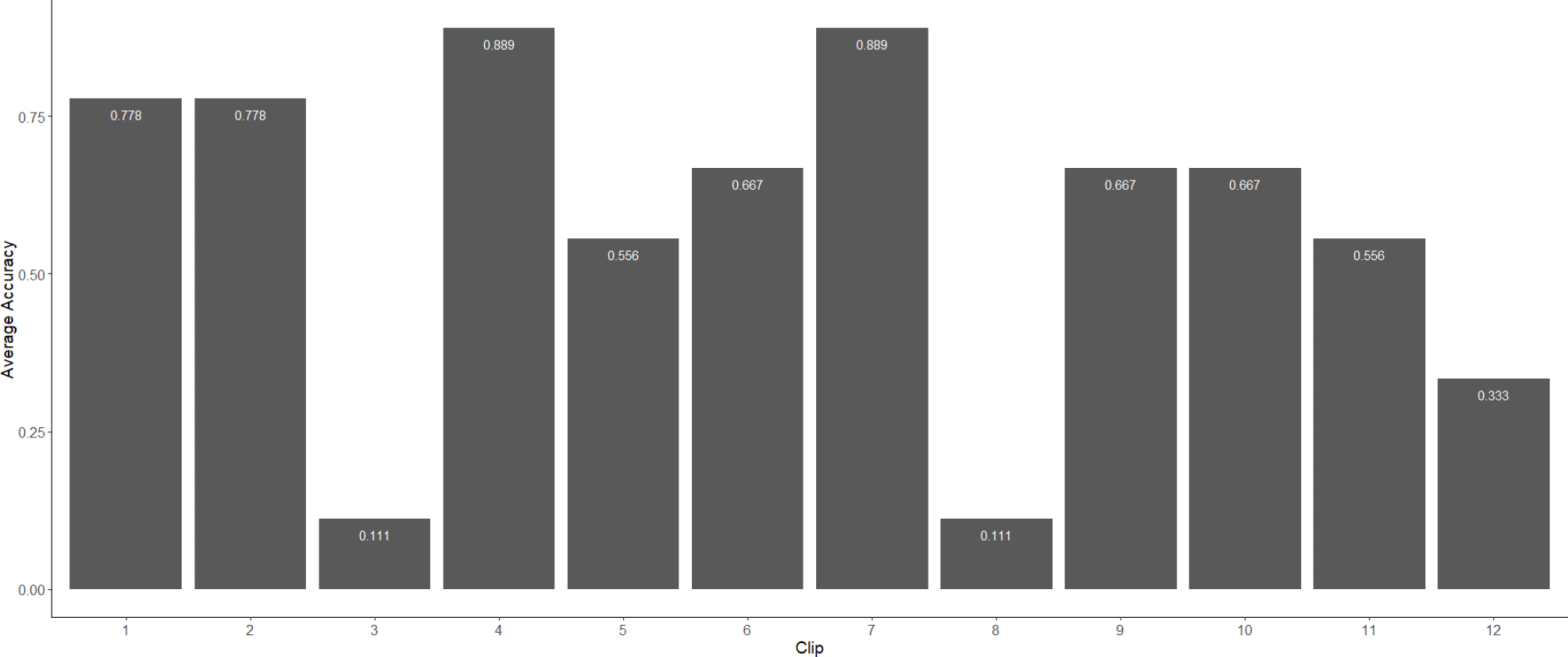
Average accuracy scores for hazard prediction across each clip during the pilot study.

The participants group consisted of one male (aged 26), six females (mean age = 23.7; SD = 4.1), and two non-binary individuals (mean age = 19.5; SD = 1.5). All participants in the pilot study were either car drivers, cyclists, or e-scooter riders, with one participant using e-scooters as their primary mode of transport. During the pilot study, participants were shown all 23 video clips following the same procedure as in the main study (see, Procedure section). Following the pilot study, participants’ responses were reviewed, and average scores were calculated for each clip to determine if any of the clips were likely to result in floor or ceiling effects based on their difficulty. This review also aimed to identify any potential issues with the multiple-choice options or instructions. Figure 4 presents the average scores for the hazard prediction clips. During the pilot study, three clips (3, 8, and 12) were identified as being either too difficult or too easy. To address these issues, the distribution of responses across the multiple-choice options was analysed. For clips 3 and 8, it was noted that participants predominantly selected only one of the distractor options, indicating a lack of plausibility in the alternatives provided. Conversely, in clip 12, the majority of participants chose a distractor over the correct answer, suggesting confusion or ambiguity in the response options. Based on this feedback, revisions were made to the distractor options, ensuring they more clearly represented different aspects of the riding scenario, particularly for clip 12, where the original distractor closely resembled the correct answer.

**Fig. 5:**
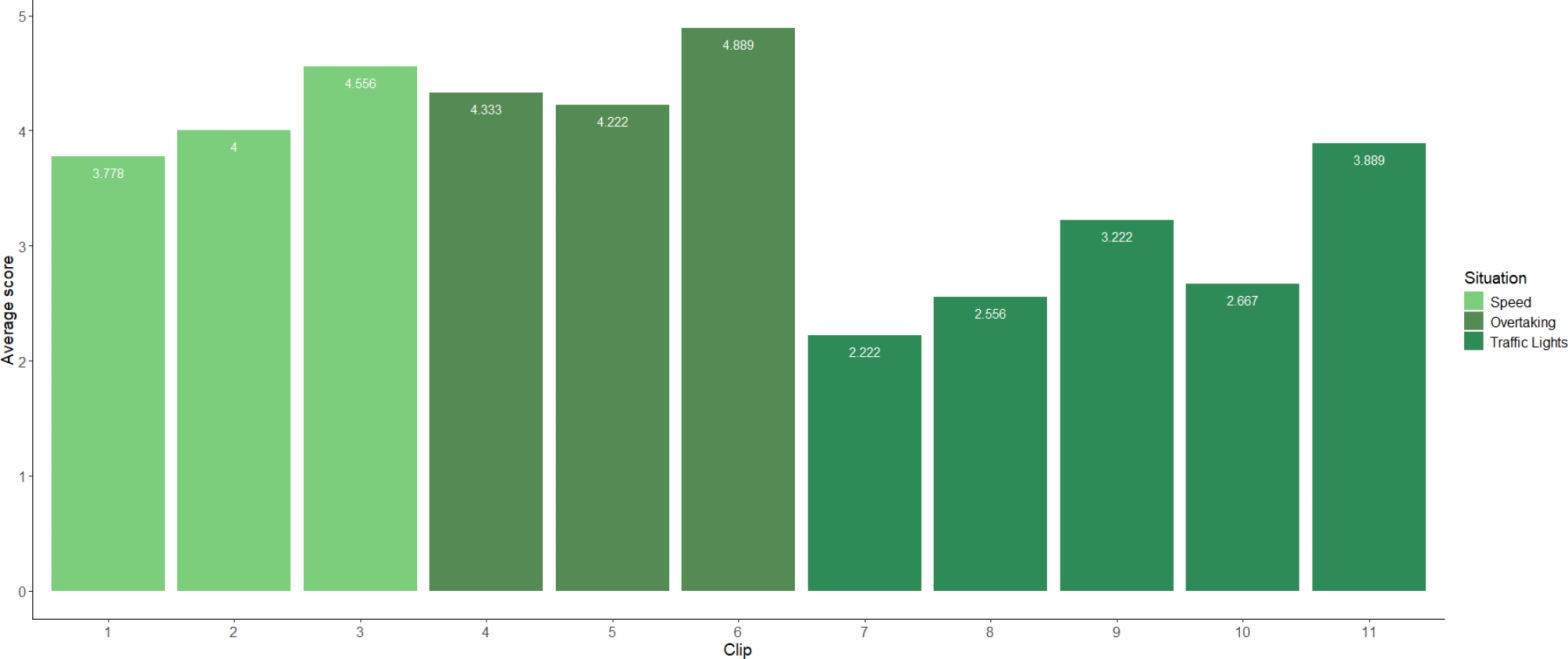
Average risk calibration ratings across each clip during the pilot study.

Figure 5 presents the average risk scores assigned to each stimulus in the risk calibration test. Although none of the video clips posed difficulty for participants, it was observed that one scenario involving an overtaking situation overlapped with another scenario used in the hazard prediction test. To address this issue, this clip was replaced with an alternative overtaking scenario.

### Procedure

This research was approved by the Ethics Committee of Nottingham Trent University (ID 1856229). The research was conducted in accordance with the Declaration of Helsinki and adhered to the ethical guidelines of Nottingham Trent University for research integrity and health and safety. All participants were thoroughly informed about the aim and procedure of the study. All participants were asked to provide consent before taking part in the study. No deception was involved, and participants were debriefed after their participation. They were also provided with information on the current e-scooter legislation.

The study was conducted online using the recruitment platform Prolific and built for online testing via Gorilla Software, allowing participants to access the experiment via their personal computers or laptops. Access to the experiment was disabled for mobile phones and tablets to ensure that participants could clearly view the videos, accurately identify the precursor to each hazard, and effectively assess each risky scenario. Upon accessing the experiment, participants were first provided with information about the study and asked to give their informed consent to participate. They then completed a short demographic questionnaire, followed by a brief road profile questionnaire to determine their experience with e-scooters and their riding habits. Participants who had never used an e-scooter were redirected to the end of the study. Following the demographic and road profile questionnaires, participants proceeded through the hazard prediction and risk calibration tests. Each test was preceded by instructions to ensure participants understood what was required of them. The order of presentation for each test block was randomized. After each task, participants were offered a short break before continuing to the next block, if needed.

For the hazard prediction task, participants were shown video clips from an e-scooter rider’s perspective and required to predict a hazardous scenario. They were instructed to watch carefully, as each clip would stop immediately prior to the hazardous situation, at which point they would be asked to predict what would happen next on the riding scene. All participants received clear and detailed instructions with a practice video to familiarise themselves with the task. They were then presented with four possible answers to select from, with only one correct option reflecting what actually happened after the clip cut to black. Upon beginning the task, participants were first shown a fixation screen for 3000ms prior to each clip. The hazard prediction test consisted of 12 video clips presented in a randomised order for each participant. Participants were not permitted to pause or re-watch the clips. For an example of a hazardous scenario, see Fig. 2.

For the risk calibration task, participants were shown clips filmed from an e-scooter rider’s perspective and asked to rate how likely they would be to engage in the risky scenario depicted in the video. Each clip ended before the situation fully materialised, preventing participants from seeing how the rider proceeded, thus requiring them to make their own decision based on the level of risk they would feel comfortable assuming. Participants watched 11 video clips, presented in a single block and in random order. Each clip was preceded by a 3000ms fixation screen. The clips fell into three categories: overtaking a vehicle, traffic lights, and speeding. For the overtaking scenarios, participants were asked to indicate how likely they would be to overtake a vehicle when it might not be safe. The traffic light clips involved situations where lights turned amber or red while the e-scooter rider was approaching them and participants were asked to decide whether they would proceed through the traffic lights in these situations. Finally, the speeding scenarios required participants to rate how likely they would be to travel at a speed higher than the legally permitted one for e-scooters on the road depicted in the clip. For each of the 11 clips, participants used a seven-point Likert scale ranging from 1 (not very likely) to 7 (very likely) to respond. All clips were presented in a randomised order for each participant. An example of a traffic light scenario is provided in Fig. 3.

Following the experimental block, participants were queried about their illegal riding behaviour, such as tandem riding, usage of mobile phones etc. while riding. Upon completing the questionnaire, participants were provided with a debrief and instructions on how to access their compensation.

## Results

### Reported illegal riding behaviour and trip motivation

**Fig. 6:**
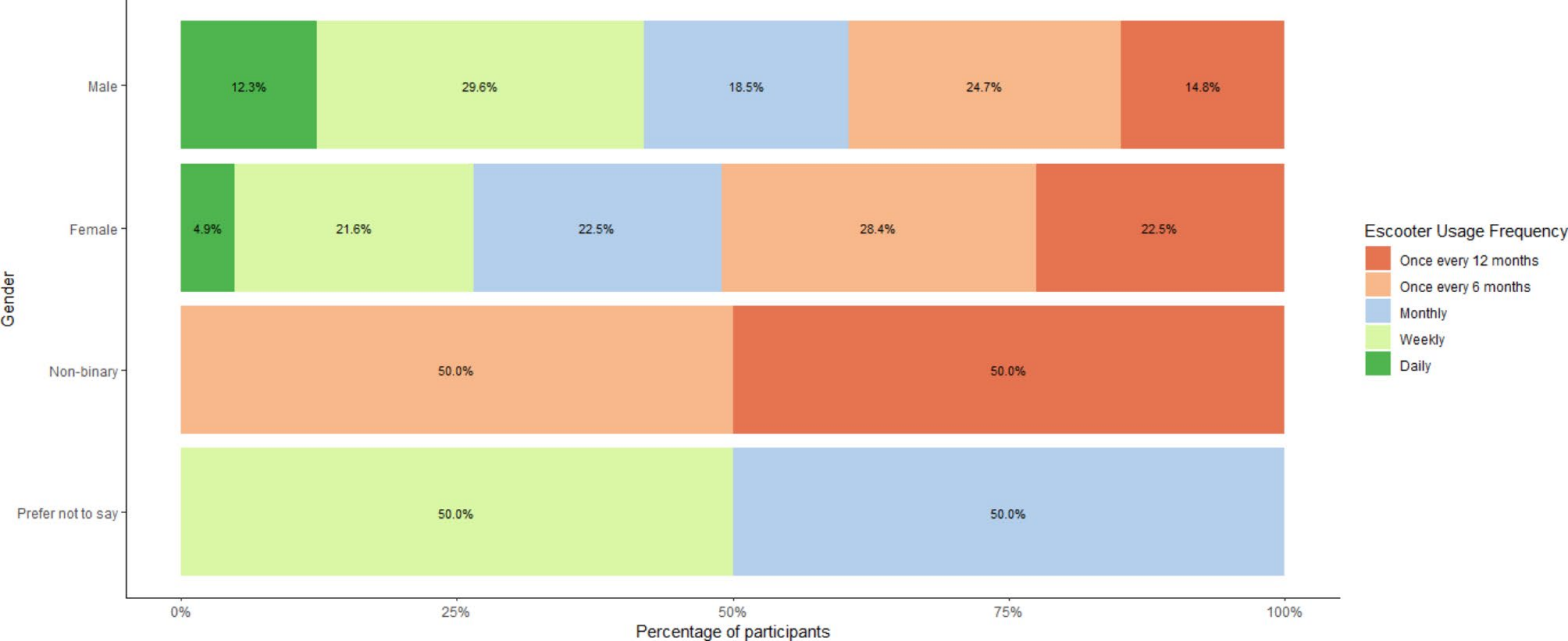
E-scooter riding frequency across gender.

Prior to the experiment, participants were asked to report their riding habits and motivations for using an e-scooter. The distribution of e-scooter riding frequency among participants is shown in Fig. 6.

**Fig. 7:**
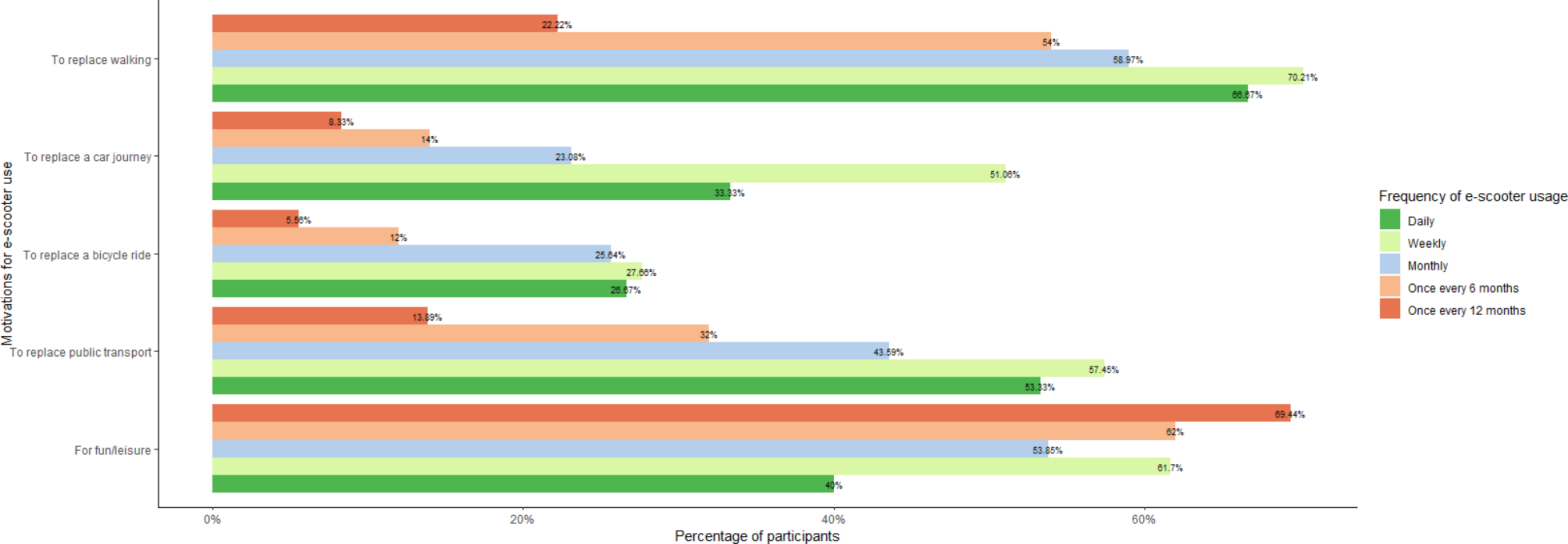
Motivation for using an e-scooter across riding frequency.

Participants were also asked to identify their motivations for using an e-scooter, with the ability to select multiple reasons. Figure 7 presents the distribution of their responses, categorised by the frequency of e-scooter use. The majority of participants indicated that their primary reasons for using e-scooters were to replace walking or for recreational purposes. Notably, the most frequent users (those who ride daily or weekly) also reported using e-scooters as alternatives to public transport and car travel.

**Fig. 8:**
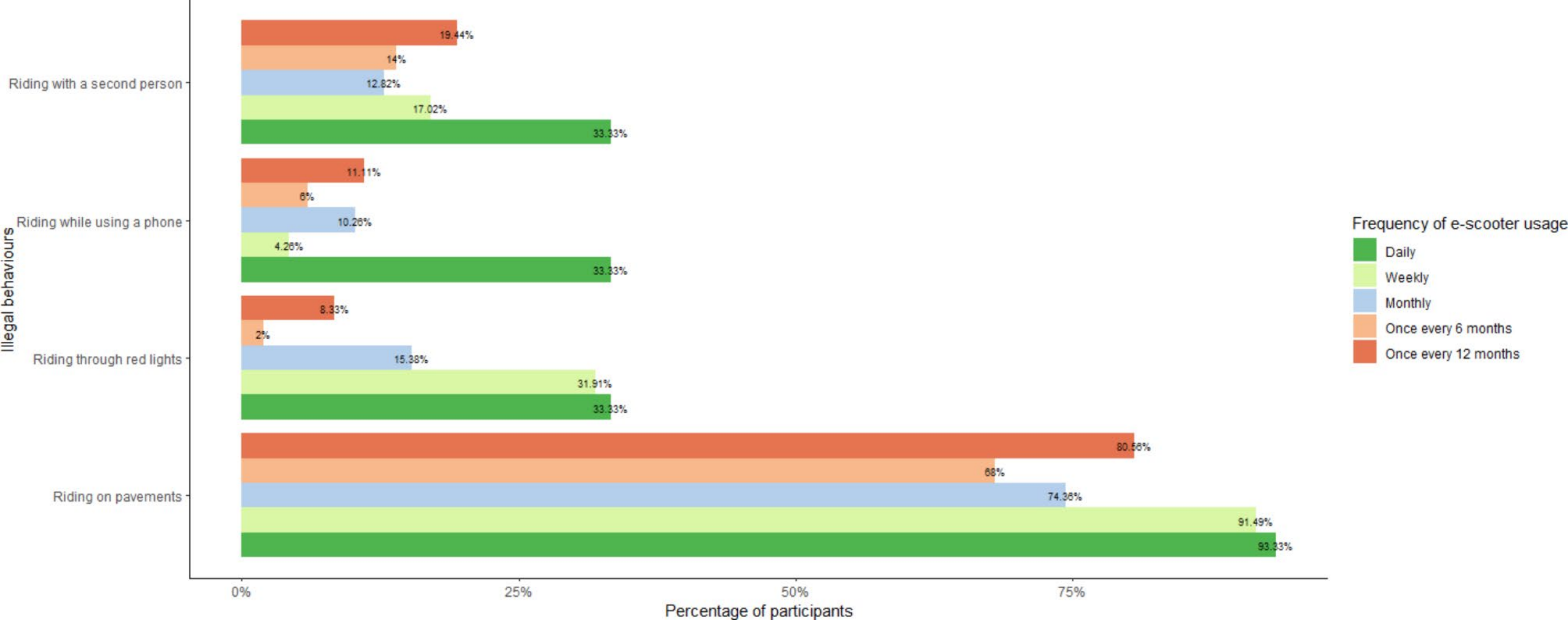
Percentage of participants who reported engaging in illegal riding behaviours across riding frequency.

Participants were also asked whether they had ever engaged in illegal riding behaviours, such as riding on the pavement, proceeding through a red light, using their phone while riding, or carrying another person on their e-scooter. Figure 8 illustrates the percentage of participants who reported engaging in these behaviours, categorised by their riding frequency. The results show that a majority of participants admitted to riding on pavements, regardless of how frequently they used an e-scooter. Additionally, a higher percentage of daily and weekly riders reported engaging in all illegal riding behaviours compared to those who ride less frequently.

### Hazard prediction accuracy and riding experience

To test for differences in hazard prediction accuracy across the selected factors, a multilevel logistic regression was performed. Participants and video clips were fitted as random factors and the experience level of participants (expressed in months and riding frequency) were treated as fixed effects. A sequence of models starting with intercept and random effects only, adding all main effects and then adding higher order interactions were conducted. Effects were tested using likelihood ratio tests to compare a model with all effects of the same order (e.g., two-way interactions) to a model where the effect of interest is dropped. Hazard prediction accuracy was coded as binary, with correct hazard prediction responses assigned a value of 1, and incorrect responses assigned a value of 0. All models were performed using the lme4 package^55^ in R Software and used a bound optimisation by quadratic approximation. Participants’ average performance in the hazard prediction task according to their e-scooter usage frequency is outlined in Table 4.

**Table 4:** Mean (and standard deviation) of hazard prediction scores according to e-scooter frequency, where a score of 1 indicated 100% accuracy and a score of 0 represents 0% accuracy derived from the raw data.

	Daily	Weekly	Monthly	Once every six months	Once every 12 months	Total
N Participants	15	47	39	50	36	187
Mean hazard prediction score	0.506(0.501)	0.450(0.498)	0.509(0.5)	0.480(0.5)	0.505(0.501)	0.487(0.500)

**Fig. 9:**
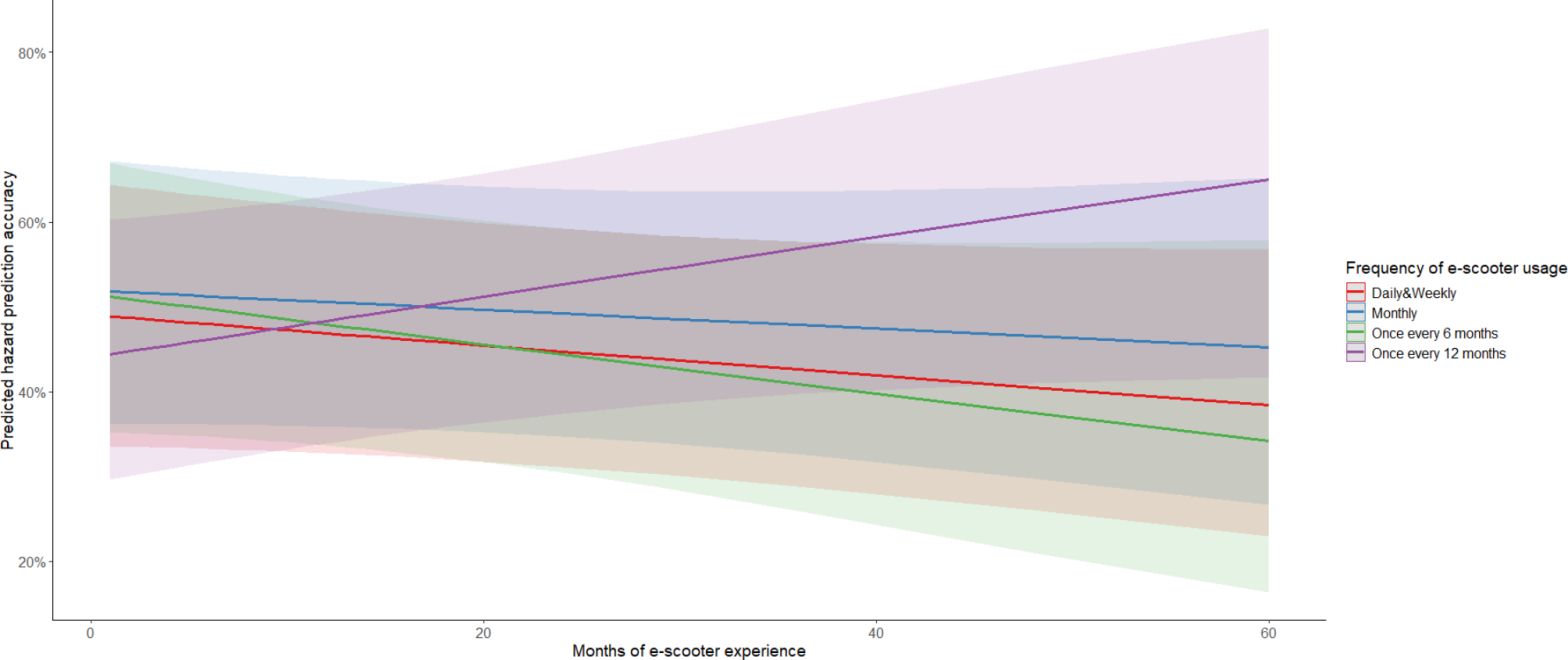
Hazard prediction accuracy scores across months of riding experience and frequency of riding.

An intercept only model (with no predictors) estimated the SD of the participant random effect as 0.28 and the SD of the clip random effect as 0.98, indicating more variability contributed to clips rather than participants. The deviance (likelihood ratio Chi Square, χ^2^) for the intercept only model was 2767.7 and decreased slightly to 2764.6 for a model including main effects of frequency and months of e-scooter usage. The intercept only model (χ^2^ = 2767.7) was a worse fit than a model with all main effects (Δχ^2^ (4) = 3.11, *p* = .54) and two-way interactions (Δχ^2^ (3) = 5.64, *p* = .13). None of the models indicated significant main effects or interactions. The number of months of e-scooter experience did not significantly predict hazard prediction accuracy (χ^2^(1) = 0.57, *p* = .45), nor did the frequency of e-scooter riding (χ^2^(3) = 2.19, *p* = .53), as shown in Fig. 9. E-scooter riders with greater months of riding experience and higher frequency of use did not demonstrate superior hazard prediction abilities compared to those with less experience and infrequent riding.

### Risk calibration skills and riding experience

To assess whether e-scooter riding experience predicts the ability to calibrate risk during risky riding scenarios, a multilevel ordinal logistic regression was performed. The video clips and participant ID were fitted as random factors and frequency and months of experience as fixed factors. The mixed models were constructed using the ordinal package in R software^56^. Effects were tested using likelihood ratio tests starting with the intercept only model and subsequently testing the main effects and interaction models. Participants’ ratings were scored so that higher ratings reflected a greater willingness to engage in risky riding behaviours. The between group factors were months of riding experience and riding frequency. The within group factor was the type of risky scenario (overtaking vs. speed vs. traffic lights). Table 5 outlines the mean scores and *SD* of participants’ performance in the risk calibration test, according to both their e-scooter usage frequency and the type of risky situation depicted.

**Table 5:** Mean (and standard deviation) risk calibration scores according to the scenario depicted and participants frequency of e-scooter usage, where a score of 7 indicates a greater likelihood to engage in risky behaviours (derived from the raw data).

	Daily	Weekly	Monthly	Once every six months	Once every 12 months	Total
N Participants	15	47	39	50	36	187
Mean risk estimation scores						
Speed	4.76(1.68)	4.76(2.01)	4.56(1.88)	4.11(1.83)	4.57(1.93)	4.508(1.91)
Overtaking	4.13(2.04)	4.87(2.01)	4.98(1.70)	4.66(1.94)	4.53(1.80)	4.713(1.90)
Traffic Lights	3.43(2.27)	3.79(2.11)	3.21(1.95)	3.24(2.05)	3.43(1.98)	3.422(2.06)
Total	3.98(2.13)	4.35(2.11)	4.06(2.02)	3.86(2.05)	4.04(1.99)	4.070(2.06)

**Fig. 10:**
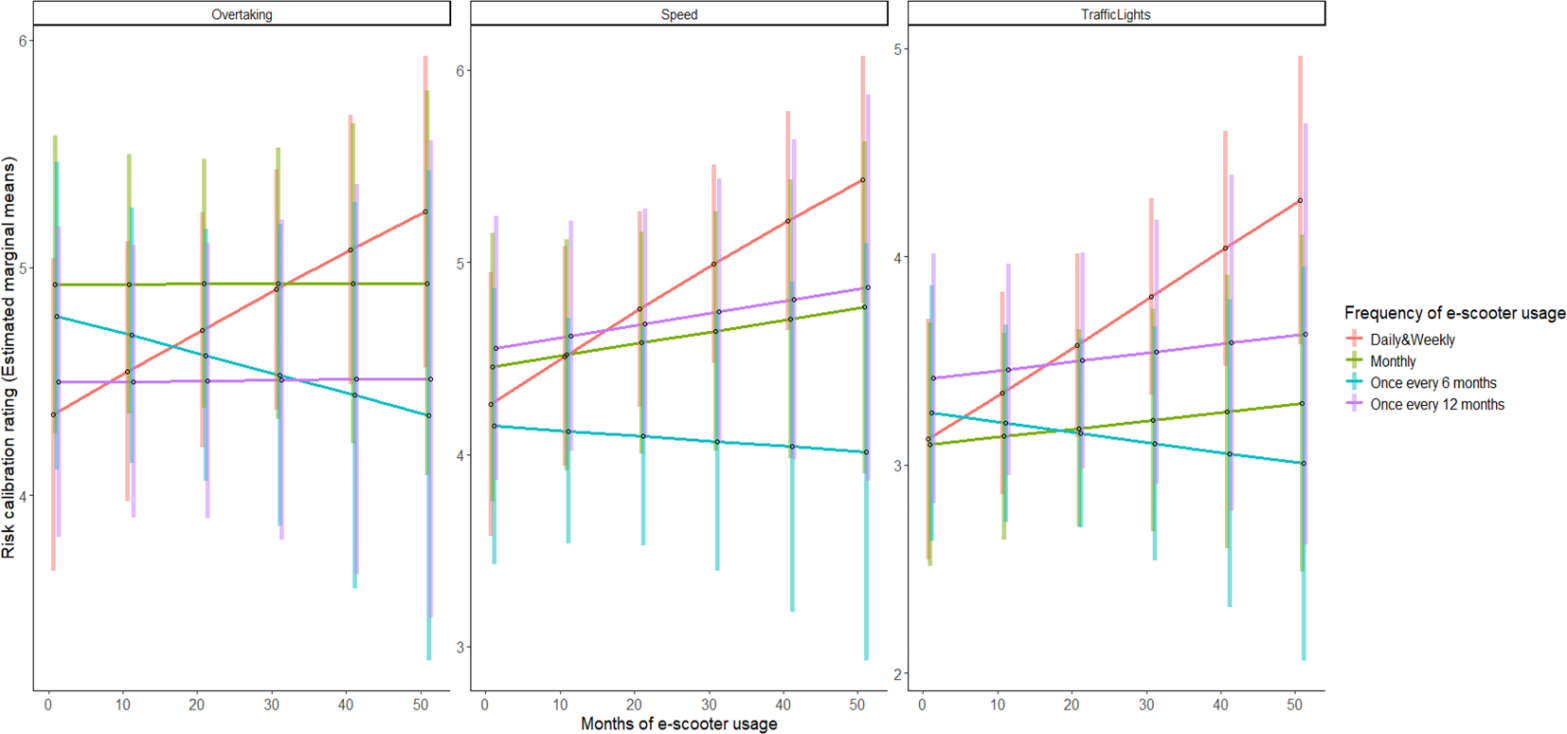
Risk calibration accuracy scores across riding frequency and months of e-scooter usage.

An intercept only model estimated the SD of the participant random effect as 0.76 and the SD of the clip random effect as 0.69, indicating slightly more variability contributed to participants. The intercept only model (χ^2^ = -3794.1) was a worse fit than a model with all main effects (Δχ^2^ (6) = 21.6, *p* = .0001) which was significant, while the model with all two-way interactions (Δχ^2^ (11) = 16.3, *p* = .13) was not. The results suggested that there was a main effect for type of risky situation χ^2^ (2) = 14.1, *p* < .0001. Participants were significantly less willing to run through red and amber traffic lights in comparison to speeding and overtaking (*p* <. 0001) (see, Table 5). There were no main effects for months and frequency of riding experience, suggesting that riding experience did not predict better risk calibration skills. However, there was a significant interaction between frequency of riding and traffic lights scenario, showing that those riders who more frequently use their e-scooters are more prone to run through red and amber traffic lights, which was especially noticeable with daily/weekly riders (*b* = -0.57; *p* <. 05) (see, Fig. 10). The interaction between months of riding experience and risky situation was not significant (Δχ^2^ (11) = 0.60, *p* = .74). The results suggested that the frequency of e-scooter use significantly predicts risk calibration albeit in the opposite direction to what was hypothesised. Figure 10 illustrates a trend indicating that more frequent riders are generally more likely to engage in risky behaviours.

## Discussion

This study aimed to assess whether e-scooter riding experience can predict improved hazard prediction and risk calibration skills over time in e-scooter riders. As this is the first study to evaluate these skills in this population, bespoke hazard prediction and risk calibration tests were developed featuring video clips from the perspective of an e-scooter rider. The tests assessed the ability of e-scooter riders to predict hazards and calibrate risk while making decisions on whether to engage in each one of the risky situations. These skills were then compared across the riders’ length and frequency of riding experience.

Surprisingly, the findings indicated that overall riding experience does not directly predict improvement in any of these skills. In other words, as riding experience and frequency increased, no corresponding improvement was observed in hazard prediction and risk calibration skills. However, a main effect for riding scenario in risk calibration was observed indicating that riders were more likely to engage in overtaking and speeding behaviours than running through red and amber traffic lights. Likewise, previous studies have reported that car drivers were found to be more likely to engage in overtaking and speeding rather than running through red and amber traffic lights^32,57^. In addition, a significant interaction was observed between riding scenario and riding frequency, though in a direction contrary to the initial hypothesis. It was anticipated that more frequent riders would be less willing to engage in risky riding behaviour, particularly at traffic lights. However, daily and weekly riders were more likely to run through red and amber lights compared to those who rode less frequently. Studies have suggested that typically, frequent driving or cycling provides individuals with the opportunity to learn how to adequately calibrate risk, leading to a decreased likelihood of engaging in dangerous situations over time^29^. However, this process with e-scooter riders appears to be more complex. Not only did increased riding experience not result in improved hazard and risk calibration skills, but it also suggested that more experienced riders may actually be more willing to assume greater risks in certain scenarios. In fact, all frequent riders in the current study reported a higher percentage of engagement in illegal riding behaviour compared to less frequent riders. A possible reason for this could be that increased riding experience leads riders to become more confident in their skills rather than more cautious, making them more inclined to engage in risky riding behaviours. The Risk Allostasis Theory^58^, a refined version of the Rrisk Homeostasis Theory, suggests that individuals establish a preferred range of risk and adjust their behaviour according to the boundaries of this range. The rapid implementation of e-scooters globally, often with insufficient focus on safety, may have contributed to a pronounced cognitive optimism bias among riders. This optimism bias, characterised by the belief that one is more skilled and less likely to encounter negative events, has been linked to increased risky behaviour when individuals perceive they have control over their environment^59,60^. Given the absence of official e-scooter training and standardised safety guidelines, there is no accepted definition of proficient e-scooter riding skills, especially cognitive ones. As a result, riders may rely on personal and subjective criteria to decide what is a risky situation and what constitutes a hazard. Given the current findings and the limited evidence regarding e-scooter riders’ ability to predict hazards and calibrate risk, further research is necessary to understand the underlying processes of how these skills are applied by riders.

The results of the present study clearly suggested that e-scooter riding experience may require more nuanced parameters for evaluation, rather than relying solely on frequency and months of use, as is common in driving studies^22,29,45^. For instance, the typically short trips undertaken with e-scooters compared to other modes of transport may have limited exposure to a variety of scenarios, which hampers the development of riders’ hazard and risk calibration skills. Even when riding is frequent during these short trips, they do not provide many opportunities to improve these skills which can explain the positive relationship between riding frequency and willingness to engage in risky riding. Notably, the relatively short time since e-scooters have been introduced may not have allowed sufficient time for riders to develop their riding experience fully. Nonetheless, it was expected that those riders with three years of riding experience would still demonstrate superior hazard prediction and risk calibration skills compared to those with less than a year of infrequent riding^46^. Since this was not the case, it is suspected that additional parameters, such as trip length and purpose, in conjunction with riding duration and frequency, may provide a better understanding of the factors associated with improved risk and hazard skills. A recent study already suggested that short e-scooter trips taken for leisure purposes are significantly associated with an increased propensity for engaging in illegal riding behaviour^61^.

It is widely recognised that e-scooters are perceived by many users as less serious and dangerous by users than other modes of transport, which can influence the level of risk riders are willing to assume when riding an e-scooter^13,62^. This has also been evidenced by both our participants’ responses and findings from other studies, which show that many e-scooter riders operate their vehicles while intoxicated, or while using their phones—behaviours they might avoid when driving a car. Key factors contributing to e-scooter crashes include intoxication, excessive speed, loss of balance, and unsafe riding behaviours^3,7,63,64^. In addition, e-scooter riders are considered to be more unpredictable than cyclists. A study conducted by Distefano et al., (2024)^12^ comparing the behaviour of cyclists and e-scooter riders at intersections found that e-scooter users approached the intersections with a higher level of risk compared to cyclists. Moreover, e-scooter trauma patients are more likely to require major trauma centre intervention compared to bicycle-related trauma patients (60.4% vs. 46.9%)^6^. While riding experience did not show an impact on risk calibration for speeding and overtaking in the present study, all participants reported a tendency to engage in risky behaviour at levels above the midpoint which is consistent with the evidence discussed above.

Riding experience did not predict better performance in hazard prediction either. However, the average hazard prediction accuracy scores of the participants did not reach 50%, indicating that their e-scooter hazard skills were below average and in need of improvement. Typically, it is expected that riders with at least one year of experience will obtain higher scores on a hazard prediction test^50^. As discussed above, overconfidence in riding skills stemming from the perception that e-scooters pose minimal danger can lead to riskier behaviours that offset any gains in hazard prediction even in experienced riders. Since e-scooter trips are typically short, riders have limited exposure to a variety of hazards. This limits their opportunity to encounter diverse situations and process different hazardous scenarios, thereby hindering their ability to build on their riding experience. This is supported by the Biased Competition Theory which equally posits that heightened abilities to anticipate contextual cues would occur through repeated exposure and practice to store in our past experiences^25^. Riders predominantly use e-scooters for short trips in city centres, often along familiar routes with predictable environments that do not adequately challenge their hazard prediction abilities.

Interestingly, one of the most effective interventions for reducing optimism bias in the driving context is hazard perception training, which has been shown to increase drivers’ awareness of their own limitations in risky situations^65^. Giving more emphasis on the skills required for safe riding and their importance can help challenge personal misconceptions and reduce optimism bias^66^. Unlike formal driving training, e-scooter use is often self-taught, lacking structured guidance on hazard identification and response. E-scooters were introduced with relatively short notice and without safety guidance or clear regulations, which likely led users to underestimate the associated risks and disregard the importance of safe riding practices^67^. This lack of safety consideration has contributed to a perception among riders that e-scooters are safer or less risky compared to other modes of transportation, resulting in diminished vigilance in hazard perception and inadequate risk calibration. Future studies should explore the potential for training these skills and evaluate which aspects of riding experience influence their improvement. It should be noted however that while there is compelling evidence supporting the effectiveness of brief computer-based training in enhancing hazard perception skills among drivers^68,69^, further research is required to draw definitive conclusions regarding its impact on reducing crash risk, particularly across different demographic groups^70^. This highlights the need for tailored hazard perception training programmes specifically designed and rigorously evaluated for e-scooter users. While hazard perception training has shown promise in driver populations, the applicability and efficacy of such interventions for e-scooter users remain largely unexplored. Such programmes should assess not only the improvement of hazard perception skills but also their potential to mitigate crash risk within this population.

The study is not without limitations. The average age of participants, which was approximately 34 years, is older than the demographic typically associated with e-scooter use, often identified as those between 16 and 25 years old. Nonetheless, other studies have found similar results in relation to risky behaviour with participants in a comparable age range of 30 to 35 years^6^. Future research should focus on examining different age groups to determine whether age would show significant links with hazard prediction and risk calibration in e-scooter riding.

In addition, participants in online studies are often prone to distractions and multitasking, which can affect the results of the study. However, research indicates that participants generally provide quality responses regardless of the device or context^71,72^. The testing platform ensured rigor by filtering out responses that did not adhere to the study’s duration and instructions, and videos could not be paused or replayed. Studies on hazard perception and risk assessment have shown that online hazard/risk skill testing and training can yield valid results^23,32,68^.

Finally, it should be noted that these findings are based on video-based tasks where participants subjectively judged their potential behaviour, rather than observing their likelihood to engage in such risky scenarios during actual riding. While e-scooter riders would still need to assess risk before executing certain actions in real-world situations, their actual behaviour on the road may differ. Nonetheless, the results of the present study offer significant insights into potential e-scooter rider behaviour, aligning with findings from other studies and government data^9,10,61^. Research has shown that perceived risk and the way individuals appraise risky situations can significantly impact actual driving behaviour^73^. Therefore, future studies should observe and analyse real-world riding behaviour to determine whether it aligns with self-reported decisions when assuming risk in specific riding situations.

In conclusion, this study represents a pioneering effort to develop tests that evaluate cognitive skills, such as hazard prediction and risk calibration, among e-scooter riders, and to assess the impact riding experience has on these skills. The findings suggested that hazard prediction and risk calibration skills do not seem to improve with riding experience over time. In fact, it was observed that more frequent e-scooter riders were more prone to engage in risky riding. This may be due to the insufficient focus on safety, which could have contributed to a pronounced cognitive optimism bias among riders. E-scooter riders may perceive themselves as more skilled and better able to control risky situations than their actual abilities allow. In addition, e-scooter experience may be influenced by factors beyond just the duration and frequency of riding including the nature of risky scenarios, trip length, and trip motivation. Short trips do not offer the same opportunities to encounter hazardous scenarios and gain experience in managing them. The high propensity for risk-taking observed among riders, coupled with their average hazard prediction scores, highlights the need for enhanced rider education and training. Such interventions are essential to improve riders’ vigilance, hazard anticipation, and adequate risk calibration in risky situations.

## Data Availability

The datasets generated during and/or analysed during the current study are available from the corresponding author on reasonable request.
